# Dietary exposure to food additives in ultra-processed foods: implications for gut microbiome, metabolic health, and risk assessment

**DOI:** 10.3389/fpubh.2026.1843650

**Published:** 2026-06-30

**Authors:** Corina Dalia Toderescu, Elena Narcisa Pogurschi, Alina Stefanache, Melania Florina Munteanu, Svetlana Trifunschi, Alexandru Oancea, Meda-Ada Bugi, Iulia Cresneac

**Affiliations:** 1 Faculty of Pharmacy, “Vasile Goldis” Western University of Arad, Arad, Romania; 2 Faculty of Animal Productions Engineering and Management, University of Agronomic Sciences and Veterinary Medicine, Bucharest, Romania; 3 Faculty of Pharmacy, Grigore T. Popa University of Medicine and Pharmacy, Iasi, Romania

**Keywords:** artificial sweeteners, dietary exposure, dietary patterns, emulsifiers, food additives, gut microbiome, metabolic health, ultra-processed foods

## Abstract

**Background:**

The increasing consumption of ultra-processed foods has led to a substantial rise in dietary exposure to food additives, making them a consistent component of modern dietary patterns. While food additives are generally considered safe within established regulatory limits, their long-term health effects remain a subject of growing scientific interest, particularly from a nutritional epidemiology perspective.

**Methods:**

This narrative review, conducted following PRISMA-informed principles, synthesizes recent experimental, clinical, and epidemiological evidence on the health effects of major food additive categories, including emulsifiers, non-nutritive sweeteners, preservatives, and synthetic colorants. Literature searches were performed in Web of Science, Scopus, and PubMed, covering studies published between 2010 and 2024. A qualitative assessment of study quality was performed based on study design, sample size, and potential sources of bias. The results of this assessment are summarized in Table 2.

**Results:**

Dietary exposure to food additives through ultra-processed foods has been associated with changes in the gut microbiome, metabolic function, and low-grade inflammation. Experimental studies consistently report biological effects, particularly for emulsifiers and artificial sweeteners, whereas epidemiological findings remain heterogeneous and influenced by overall dietary patterns.

**Conclusion:**

Current evidence supports the need to evaluate food additives within the context of dietary patterns rather than as isolated compounds. While most approved additives remain safe at regulated intake levels, emerging data suggest that cumulative exposure and diet–microbiome interactions may not be fully captured by existing risk assessment frameworks. Integrating nutritional context into future safety evaluations may improve their relevance for public health.

## 1 Introduction

Food additives are integral components of modern food systems. They are widely consumed through processed and ultra-processed foods that increasingly define contemporary dietary patterns (1, 2). The global shift toward industrially formulated foods has increased chronic exposure to food additives. These are now a routine part of daily intake rather than occasional components of the diet (2). Unlike traditional regulatory evaluations, which primarily assess food additives as isolated compounds based on acceptable daily intake thresholds, this review adopts a systems-level perspective. It integrates dietary exposure within ultra-processed food patterns and highlights microbiome-mediated mechanisms as potential pathways linking additive consumption to health outcomes. This approach provides a complementary framework that may better reflect real-world exposure conditions and inform future risk assessment strategies.

From a nutritional perspective, the health relevance of food additives must be considered within the context of dietary patterns in which they are consumed. Ultra-processed foods represent a major source of additive exposure and have been consistently associated with adverse health outcomes, including obesity, metabolic disorders, and chronic disease risk (3, 4). This raises important questions about whether additive exposure contributes to these associations. These effects may occur independently or through interactions with overall diet quality.

Although food additives are generally considered safe within established regulatory limits, based on toxicological assessments and acceptable daily intake thresholds, emerging evidence suggests that their biological effects may not be fully captured by traditional safety frameworks (1, 5). In real-world conditions, additives are consumed chronically, in combination, and within complex dietary matrices, which challenges the relevance of single-compound risk assessment approaches.

Recent advances in microbiome research and nutritional epidemiology have provided new insights into potential mechanisms linking dietary exposure to food additives with human health outcomes (6–8). Studies suggest that certain additives, particularly emulsifiers and non-nutritive sweeteners, may influence gut microbiome composition, intestinal barrier integrity, metabolic regulation, and inflammatory responses (9–12). These findings highlight the importance of considering diet-microbiome interactions when evaluating additive-related health effects.

Despite growing evidence, important limitations remain. Many studies investigate individual additives under controlled conditions, whereas real-world exposure occurs through dietary patterns characterized by the simultaneous intake of multiple additives (13, 14). In addition, epidemiological research faces challenges in accurately assessing additive intake and disentangling additive-specific effects from those of ultra-processed food consumption.

The objective of this review is to critically evaluate current evidence on the health effects of dietary exposure to food additives, with particular emphasis on their role within ultra-processed dietary patterns and their implications for metabolic, gastrointestinal, and microbiome-related outcomes.

## 2 Methods

### 2.1 Study design

This narrative review was conducted following PRISMA guidelines where applicable. This included database searching, screening, and eligibility assessment.

### 2.2 Data sources and search strategy

A structured literature search was performed in three major scientific databases: Web of Science, Scopus, and PubMed/MEDLINE. The search covered studies published between January 2010 and December 2024 and was limited to peer-reviewed articles published in English.

### 2.3 Search terms

were developed to reflect both food additive categories and relevant health outcomes, as well as their dietary context. The following keyword combinations were used:

“food additives and health”

“dietary exposure and food additives”

“ultra-processed foods and additives”

“artificial sweeteners and metabolism”

“emulsifiers and gut microbiome”

“food colorants and neurobehavior”

“preservatives and chronic disease”

“food additives and inflammation”

Boolean operators and database-specific filters were applied to refine the search and exclude irrelevant records.

**Figure 1:**
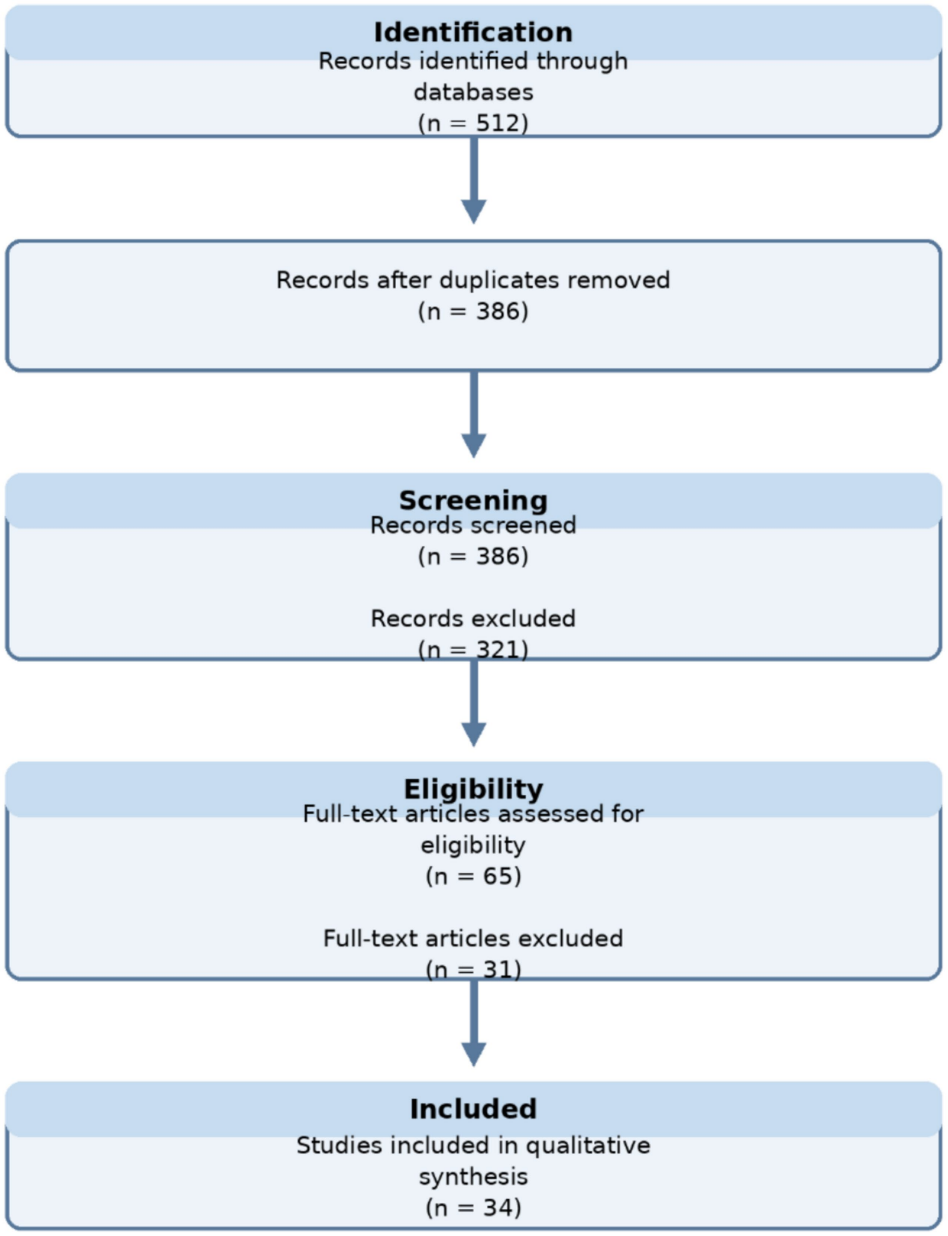
PRISMA flow diagram illustrating the study selection process, including identification, screening, eligibility, and inclusion of studies.

The study selection process is summarized in a PRISMA flow diagram (Figure 1 illustrates these pathways). A total of 512 records were initially identified through database searches. After removal of duplicates, 386 records were screened, of which 321 were excluded based on title and abstract. A total of 65 full-text articles were assessed for eligibility, and 34 studies were included in the final synthesis.

### 2.4 Eligibility criteria

Studies were included if they met the following criteria: peer-reviewed articles human, animal, or *in vitro* studies examining health effects of food additives experimental, clinical, or epidemiological study designs publications between 2010 and 2024 studies addressing metabolic, gastrointestinal, microbiome-related, neurobehavioral, or toxicological outcomes.

Studies were excluded if they: lacked primary data or scientific analysis focused exclusively on food technology without health outcomes were conference abstracts, editorials, or opinion papers were not available in full-text format.

### 2.5 Study selection

Study selection followed PRISMA-informed procedures. Records identified through database searches were screened based on titles and abstracts, followed by full-text assessment of potentially relevant articles. Duplicate records were removed prior to screening. Final inclusion was based on relevance to the study objective, focusing on evidence linking dietary exposure to food additives with human health outcomes.

### 2.6 Data extraction and synthesis

Data extraction focused on key study characteristics, including: author and publication year additive category study design population/sample exposure assessment health outcomes main findings.

Findings were synthesized narratively and organized according to additive categories and major physiological domains. Human studies were prioritized, while experimental and mechanistic studies were used to support biological plausibility.

A qualitative assessment of study quality was conducted based on study design, sample size, and potential sources of bias. Randomized controlled trials and large cohort studies were considered to have lower risk of bias, while smaller experimental studies and observational designs were classified as having moderate risk. This approach was used to provide contextual interpretation of the evidence.

### 2.7 Methodological considerations

Given the heterogeneity of study designs, exposure assessments, and outcome measures, a quantitative meta-analysis was not performed. Instead, a qualitative synthesis approach was adopted.

Although PRISMA principles were followed to enhance transparency, the review was not prospectively registered.

Recent studies published close to the time of manuscript submission were included to ensure up-to-date coverage of the topic.

Table 1 summarizes the main characteristics of the human studies included in this review. Overall, the evidence indicates that emulsifiers and artificial sweeteners are the most frequently investigated additives, with reported effects primarily involving metabolic outcomes, gut microbiome alterations, and gastrointestinal responses.

**Table 1:** Characteristics of key human studies investigating dietary exposure to food additives and health outcomes.

Author, year	Additive category	Study type	Population/sample	Exposure assessment	Main outcomes	Direction of findings
Suez et al., 2014 (10)	Artificial sweeteners	Experimental human study	Adults	Dietary intake + microbiome analysis	Glucose tolerance	Negative metabolic effect
Chassaing et al., 2022 (9)	Emulsifiers	Randomized controlled trial	Healthy adults	Controlled dietary exposure	Gut microbiome, inflammation	Negative effect
Naimi et al., 2021 (11)	Emulsifiers	Observational study	Adult	Dietary exposure models	Microbial composition	Negative effect
Hasenböhler et al., 2026 (61)	Preservatives	Prospective cohort	NutriNet-Santé adults	Food additive intake database	Type 2 diabetes risk	Possible association
Hasenböhler et al., 2026 (15)	Preservatives	Prospective cohort	General population	Dietary records	Cancer incidence	Positive association
Chazelas et al., 2022 (62)	Nitrites/nitrates	Cohort	Adults	Dietary intake modeling	Cancer risk	Positive association
Toews et al., 2019 (16)	Artificial sweeteners	Systematic review/meta-analysis	Multiple populations	Intake data synthesis	Cardiometabolic outcomes	Mixed findings
Romo-Romo et al., 2016 (26)	Artificial sweeteners	Clinical trial synthesis	Adults	Intervention studies	Weight, glucose metabolism	Mixed findings
Sylvetsky et al., 2018 (45)	Artificial sweeteners	Review	Adults/children	Consumption patterns	Metabolic outcomes	Mixed findings
Dunford et al., 2025 (17)	Synthetic dyes	Observational	Packaged food exposure	Product database	Population exposure	High exposure prevalence
Stevens et al., 2011 (29)	Synthetic colorants	Clinical/behavioral	Children	Diet-based exposure	ADHD symptoms	Possible effect
Witkowski et al., 2022 (48)	Multiple additives	Review	Mixed populations	Reported reactions	Hypersensitivity	Possible effect
Oda et al., 2023 (63)	Phosphates	Human/experimental integration	Adults	Dietary intake + biomarkers	Gut barrier function	Negative effect
Zhou et al., 2023 (24)	Mixed additives	Review	Multiple populations	Dietary exposure	Microbiome composition	Negative effect
Lavrinienko et al., 2025 (64)	Mixed additives	Critical review	Human studies	Exposure modeling	Gut microbiome, metabolism	Mechanistic evidence
Bancil et al., 2021 (12)	Emulsifiers	Review	Human + mechanistic	Dietary patterns	Intestinal inflammation	Positive association

## 3 Results

### 3.1 Overview of included evidence

The studies included in this review indicate that dietary exposure to food additives, primarily through ultra-processed food consumption, is associated with a range of biological effects involving gastrointestinal, metabolic, microbiome-related, and neurobehavioral outcomes (9, 10, 15, 16). Most human studies have focused on emulsifiers and non-nutritive sweeteners, while evidence on preservatives, synthetic colorants, and emerging additives remains more limited (17–19). Overall, the included studies demonstrated moderate methodological quality, with lower risk of bias observed in large cohort studies and meta-analyses, and higher variability in smaller experimental and observational studies. A summary of study quality and risk of bias is presented in Table 2.

**Table 2:** Quality assessment of included human studies.

Study	Study design	Sample size	Risk of bias
Chassaing et al., 2022 (9)	RCT	Small	Moderate
Suez et al., 2014 (10)	Experimental/human	Small	Moderate
Toews et al., 2019 (16)	Meta-analysis	Large	Low
Chazelas et al., 2022 (62)	Cohort	Large	Moderate
Hasenböhler et al., 2026 (15, 61)	Cohort	Large	Moderate
Azad et al., 2017 (27)	Meta-analysis	Large	Low

Risk of bias was qualitatively assessed based on study design, sample size, and methodological limitations. RCT, randomized controlled trial.

### 3.2 Gastrointestinal effects

Evidence from experimental and clinical studies suggests that dietary exposure to certain food additives may influence gastrointestinal physiology and intestinal homeostasis. Emulsifiers, artificial sweeteners, and selected preservatives have been associated with alterations in intestinal barrier integrity, mucus layer composition, and immune signaling (11, 12, 20).

Experimental models consistently demonstrate that emulsifiers can disrupt gut barrier function, promote low-grade inflammation, and modify host-microbiome interactions (21). Although these effects are often observed under controlled conditions, they provide biologically plausible mechanisms through which additive exposure may contribute to gastrointestinal dysfunction (22, 23).

### 3.3 Gut microbiome alterations

The gut microbiome has emerged as a key mediator of the effects of dietary exposure to food additives. Multiple studies report that emulsifiers, artificial sweeteners, and certain preservatives can alter microbial composition, diversity, and metabolic activity (9–11, 24, 25).

Artificial sweeteners have been shown to induce microbiome-mediated metabolic changes, including glucose intolerance in experimental settings, while emulsifiers have been associated with dysbiosis and pro-inflammatory microbial profiles (9, 11, 12). Similarly, randomized controlled feeding studies have demonstrated that exposure to specific emulsifiers can significantly alter gut microbiome composition and metabolic markers in humans. These findings suggest that microbiome alterations may represent a central pathway linking dietary additive exposure to host metabolic and inflammatory responses.

### 3.4 Metabolic effects

Emerging evidence indicates that dietary exposure to food additives may be associated with metabolic outcomes such as insulin resistance, altered glucose metabolism, and obesity-related pathways (16, 26, 27). For example, in a large prospective cohort study from the NutriNet-Santé population, higher intake of certain food additive preservatives was associated with an increased risk of type 2 diabetes, with hazard ratios indicating a statistically significant positive association between exposure and disease incidence. Non-nutritive sweeteners have been the most extensively studied, with findings ranging from neutral effects to potential metabolic dysregulation (28).

Emulsifiers and certain preservatives have also been implicated in metabolic disturbances, potentially through inflammation and microbiome-mediated mechanisms (10, 20). However, epidemiological evidence remains heterogeneous, reflecting differences in study design, exposure assessment, and dietary context (13, 16).

### 3.5 Neurobehavioral effects

Some studies suggest that synthetic food colorants and other additives may influence neurobehavioral outcomes, particularly in children. Associations with hyperactivity-related behaviors have been reported (19, 29, 30), although findings remain inconsistent and context-dependent.

Proposed mechanisms include interactions with neurotransmitter systems, oxidative stress pathways, and immune signaling (18, 31). However, evidence in human populations remains limited and does not allow firm conclusions.

### 3.6 Long-term health outcomes

Long-term dietary exposure to food additives has been investigated in relation to chronic diseases, including cancer, cardiovascular disease, and metabolic disorders. Prospective cohort studies suggest potential associations between certain additives, particularly preservatives, and disease risk, although causality remains difficult to establish (4, 15).

A major limitation of current evidence is the difficulty of disentangling additive-specific effects from those of overall dietary patterns, especially in populations with high consumption of ultra-processed foods (3, 13, 14).

## 4 Discussion

This review highlights the growing complexity of evaluating the health effects of food additives within modern dietary patterns. While traditional risk assessment frameworks consider additives safe within established intake limits, emerging evidence suggests that their biological effects may depend on cumulative exposure, dietary context, and interactions with host physiology (32, 33).

One of the central findings of this review is the heterogeneity of available evidence (34). Experimental studies consistently demonstrate biological effects of certain additives, particularly emulsifiers and non-nutritive sweeteners, on gut microbiome composition, intestinal barrier function, and inflammatory pathways (12, 24, 35). In contrast, epidemiological findings in human populations remain inconsistent. This discrepancy likely reflects differences in exposure levels, study design, and the inherent challenges of isolating additive-specific effects within complex dietary patterns (36, 37).

Additive mixtures and cumulative exposure.

In real-world dietary contexts, food additives are rarely consumed in isolation but rather as part of complex mixtures within ultra-processed foods. This raises important questions regarding potential cumulative and synergistic effects that are not fully captured by traditional risk assessment frameworks. While current regulatory approaches typically evaluate additives individually, emerging evidence suggests that combined exposures may produce additive or interactive biological effects, particularly at the level of the gut microbiome and immune system.

The concept of mixture toxicity highlights the possibility that multiple additives, even at levels considered safe individually, may collectively influence metabolic and inflammatory pathways. However, the current evidence base on additive mixtures remains limited, and most studies focus on single compounds under controlled conditions. This gap underscores the need for more research investigating real-world exposure scenarios and the combined effects of multiple additives within dietary patterns.

While additive mixtures are often discussed in terms of cumulative or synergistic effects, antagonistic interactions may also occur and should be considered in dietary risk assessment. Certain food additives may attenuate or counterbalance the biological activity of others through mechanisms involving competition for metabolic pathways, altered bioavailability, or modulation of oxidative and inflammatory responses. Examples of synergistic interactions include combined effects of emulsifiers and dietary components on gut barrier integrity and microbiome composition, whereas antagonistic interactions may occur when compounds with antioxidant properties reduce oxidative processes induced by other dietary constituents. These findings highlight that the health effects of additive mixtures cannot always be predicted from the evaluation of individual compounds alone (33, 38, 39).

Despite the growing body of evidence, findings remain heterogeneous across study types. Experimental studies, including animal models and controlled feeding trials, often isolate specific additives under tightly controlled conditions, allowing for the identification of direct biological effects. In contrast, epidemiological studies reflect real-world dietary patterns, where exposure occurs through complex mixtures of additives and is influenced by multiple lifestyle and environmental factors. Differences in exposure levels, variability in dietary assessment methods, and the presence of confounding variables further contribute to the divergence between experimental and human findings. Additionally, experimental conditions may involve higher or more controlled doses than those typically encountered in habitual diets, which may limit direct translation to population-level outcomes. An important limitation of the current evidence relates to the challenge of establishing causality. Observational studies are particularly susceptible to confounding, as individuals with higher consumption of ultra-processed foods often exhibit multiple concurrent lifestyle and dietary risk factors, including lower overall diet quality, reduced physical activity, and socioeconomic influences. In addition, reverse causality may influence observed associations, as individuals with existing metabolic or health conditions may modify their dietary habits, including increased consumption of certain additive-containing foods such as low-calorie or “diet” products. These factors complicate the interpretation of epidemiological findings and highlight the need for cautious interpretation of associations between dietary additive exposure and health outcomes.

### 4.1 Differential vulnerability across populations

The potential health effects of dietary exposure to food additives may not be uniform across populations. Certain groups may exhibit increased vulnerability due to physiological, genetic, or environmental factors. For example, children may be more susceptible due to lower body weight and developing metabolic and immune systems, while individuals with pre-existing metabolic or gastrointestinal conditions may experience amplified responses to additive exposure. Variability in gut microbiome composition may further influence individual susceptibility, contributing to heterogeneous responses observed across studies.

In addition, differences in dietary patterns, including higher consumption of ultra-processed foods in certain populations, may result in disproportionately higher additive exposure. These considerations highlight the importance of incorporating population-specific factors into both research and risk assessment frameworks.

### 4.2 Socioeconomic disparities in dietary additive exposure

Dietary exposure to food additives is closely linked to broader socioeconomic factors that influence food choices and access. Individuals from lower socioeconomic backgrounds often rely more heavily on ultra-processed foods due to their affordability, availability, and convenience. As a result, these populations may experience disproportionately higher exposure to multiple food additives.

This disparity raises important public health considerations, as increased additive exposure may compound existing health inequalities associated with diet quality, access to fresh foods, and lifestyle factors. Addressing these inequities requires not only improved risk assessment frameworks but also broader food system interventions aimed at promoting access to healthier dietary options across diverse populations.

### 4.3 Future research priorities

Future research should prioritize longitudinal human studies with improved methods for assessing dietary exposure to food additives within real-world dietary patterns. The development of standardized food composition databases that include detailed information on additive content would enhance the accuracy of exposure assessment. In addition, studies investigating the combined effects of multiple additives are needed to better reflect habitual dietary conditions.

Accurate assessment of dietary exposure to food additives remains a significant methodological challenge. Current approaches rely heavily on self-reported dietary data and food composition databases, which often lack detailed and standardized information on additive content. In addition, variability in food formulations and labeling practices further complicates exposure estimation.

Improving exposure assessment will require the development of comprehensive and standardized databases that capture additive content across food products, as well as the integration of objective biomarkers where feasible. Combining dietary assessment methods with digital tools and real-time data collection may also enhance the precision of exposure measurement in future studies.

Integrating microbiome and metabolomic analyses into epidemiological research may provide deeper insights into the biological mechanisms underlying observed associations. Furthermore, controlled human intervention studies using realistic exposure levels are essential to bridge the gap between experimental and observational findings. Finally, incorporating population-specific factors, including genetic variability and baseline microbiome composition, will be important for understanding differential susceptibility to additive exposure.

Dietary exposure to food additives is closely linked to the consumption of ultra-processed foods, which represent a major component of modern dietary patterns. Ultra-processed foods are characterized not only by their nutritional composition but also by the presence of multiple additives that contribute to palatability, texture, and shelf life. Emerging evidence suggests that the health effects associated with ultra-processed food consumption may not be explained solely by macronutrient content, but also by the cumulative exposure to additives and food processing-related factors. This perspective supports the need to evaluate food additives within the broader framework of dietary patterns and food system characteristics rather than as isolated compounds (2, 40).

From a nutritional perspective, these findings underscore the importance of considering dietary exposure to food additives within the broader context of ultra-processed food consumption. In real-world settings, additives are rarely consumed in isolation. They are embedded within dietary patterns characterized by multiple simultaneous exposures (13, 14, 41). This makes it difficult to disentangle the independent effects of additives from those of overall diet quality, yet it also suggests that additive-related effects may contribute to the health risks associated with ultra-processed diets.

**Figure 2:**
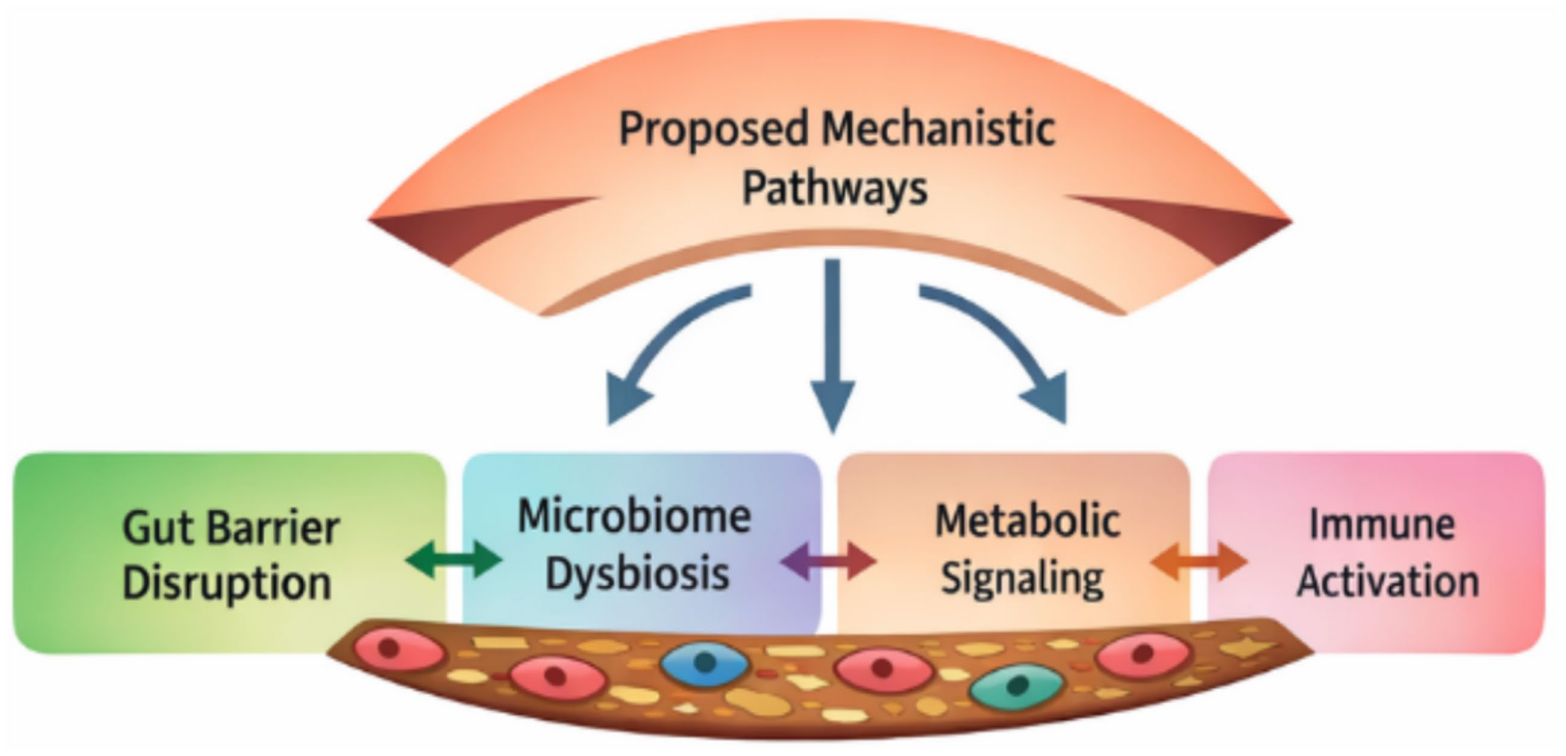
Proposed mechanistic pathways linking dietary exposure to food additives with health outcomes.

Mechanistic evidence provides important insights into how dietary exposure to food additives may influence human health. Proposed pathways include microbiome dysbiosis, disruption of intestinal barrier integrity, immune activation, and alterations in metabolic signaling. These interconnected mechanisms support a model in which additives interact with diet-microbiome-host systems. They do not act as isolated toxic agents (Figure 2) (7, 8, 42).

Food additives, primarily consumed through ultra-processed foods, may contribute to gut barrier disruption, microbiome dysbiosis, immune activation, and alterations in metabolic signaling. These interconnected pathways may collectively influence chronic disease risk.

Beyond these individual pathways, the gut microbiome plays a central integrative role in mediating the effects of dietary exposure to food additives. The gut microbiome is increasingly recognized as a central mediator linking dietary exposures to host health outcomes. Rather than acting as direct toxic agents, food additives may influence host physiology indirectly through microbiome-dependent mechanisms. Alterations in microbial composition and function can affect metabolic regulation, immune responses, and barrier integrity, highlighting the importance of considering microbiome-mediated pathways in nutritional risk assessment. This systems-based perspective aligns with emerging concepts in precision nutrition and personalized dietary responses (43, 44).

Among the different additive categories, emulsifiers and non-nutritive sweeteners show the most consistent biological signals across experimental studies. Their effects on gut microbiota composition and metabolic regulation have been repeatedly demonstrated, although translation to long-term clinical outcomes remains uncertain (45–47). Evidence regarding preservatives and synthetic colorants is more limited and often context-dependent, highlighting the need for more targeted research (31, 48, 49).

Unexpected interactions may also occur when food additives are consumed within complex dietary environments containing redox-active trace metals such as iron and copper. Under specific conditions, compounds with antioxidant properties may exhibit context-dependent behavior and influence oxidative processes through redox-mediated mechanisms. These interactions could modify biological responses, including oxidative stress signaling and microbiome-related pathways, in ways that are not captured by conventional single-compound safety evaluations. Although current evidence remains limited, such interactions represent an emerging area of interest for future dietary exposure and risk assessment research (32, 38).

Several methodological limitations should be considered when interpreting the current evidence (50). Another limitation relates to potential publication bias, as studies reporting adverse effects of food additives may be more likely to be published than studies with null findings. This may influence the overall interpretation of the available evidence. Many studies rely on animal or *in vitro* models, and human data remain limited, particularly for long-term exposure. In this review, human studies were prioritized in the interpretation of findings, while experimental studies were primarily used to support mechanistic understanding. In addition, accurate quantification of dietary additive intake is challenging, especially in observational studies where exposure is closely linked to overall dietary patterns (37, 51). The effects of additive mixtures and cumulative exposure are also insufficiently addressed in current research (33, 38, 52).

These limitations have important implications for risk assessment. Existing regulatory frameworks are primarily based on single-compound evaluations and toxicological thresholds, which may not fully capture the complexity of real-world dietary exposure. Integrating microbiome research, longitudinal epidemiology, and systems-based approaches into risk assessment models may improve their relevance for modern diets (32, 53, 54).

An additional challenge in evaluating the health effects of food additives is the real-world context of exposure, which involves complex mixtures rather than single compounds. Individuals consuming ultra-processed diets are simultaneously exposed to multiple additives, raising concerns about potential synergistic or cumulative effects. Traditional toxicological approaches, which focus on individual substances, may therefore underestimate the biological impact of combined exposures. Integrating mixture toxicology and dietary exposure assessment represents an important step toward improving the relevance of current risk evaluation models (32, 39).

From a public health and nutrition perspective, the role of food additives should be considered within broader food system dynamics. Additives contribute to the availability, affordability, and stability of ultra-processed foods, which are widely consumed in contemporary diets. However, their potential involvement in diet-related chronic diseases highlights the need for strategies that address both additive exposure and overall dietary quality (55, 56).

Current evidence suggests that the effects of dietary exposure to food additives may be mediated through several interconnected biological pathways. These include disruption of intestinal barrier integrity, microbiome dysbiosis, immune activation, and alterations in metabolic signaling. Together, these mechanisms support a systems-level model in which food additives interact with diet-microbiome-host pathways, potentially contributing to chronic disease risk (57–59).

Future research should prioritize longitudinal human studies with improved exposure assessment, investigation of additive mixtures, and integration of microbiome and metabolomic data. Such approaches will be essential to clarify causal relationships and inform evidence-based regulatory and nutritional recommendations (60).

## 5 Conclusion

Food additives are an integral component of modern dietary patterns, primarily through their widespread presence in ultra-processed foods. While most approved additives are considered safe within established regulatory limits, emerging evidence suggests that their health effects may depend on cumulative dietary exposure and interactions with the gut microbiome and host metabolism.

Current findings highlight the need to move beyond single-compound toxicological models and to evaluate food additives within the broader context of dietary patterns. Integrating nutritional epidemiology, microbiome research, and real-world exposure scenarios into risk assessment frameworks may improve their relevance for contemporary diets and public health.

Future risk assessment approaches should also consider the potential for synergistic, antagonistic, and context-dependent interactions among food additives and other dietary components, particularly under real-world exposure conditions.
